# Emergency Consent: Patients’ and Surrogates’ Perspectives on Consent for Clinical Trials in Acute Stroke and Myocardial Infarction

**DOI:** 10.1161/JAHA.118.010905

**Published:** 2019-01-19

**Authors:** Neal W. Dickert, Victoria M. Scicluna, Opeolu Adeoye, Dominick J. Angiolillo, James C. Blankenship, Chandan M. Devireddy, Michael R. Frankel, Sara F. Goldkind, Gautam Kumar, Yi‐An Ko, Andrea R. Mitchell, Raul G. Nogueria, Ruth M. Parker, Manesh R. Patel, Michele Riedford, Robert Silbergleit, Candace D. Speight, Ilana Spokoyny, Kevin P. Weinfurt, Rebecca D. Pentz

**Affiliations:** ^1^ Division of Cardiology Department of Medicine Emory University School of Medicine Atlanta GA; ^2^ Department of Neurology Emory University School of Medicine Atlanta GA; ^3^ Winship Cancer Institute Emory University School of Medicine Atlanta GA; ^4^ University of Michigan School of Medicine Ann Arbor MI; ^5^ Department of Emergency Medicine University of Cincinnati College of Medicine Cincinnati OH; ^6^ Division of Cardiology Department of Medicine University of Florida College of Medicine—Jacksonville Jacksonville FL; ^7^ Department of Cardiology Geisinger Medical Center Danville PA; ^8^ Research and Clinical Bioethics Consultant Goldkind Consulting LLC Potomac MD; ^9^ Atlanta VA Medical Center Decatur GA; ^10^ Department of Biostatistics and Bioinformatics Emory University Rollins School of Public Health Atlanta GA; ^11^ Department of Medicine Emory University School of Medicine Atlanta GA; ^12^ Division of Cardiology Department of Medicine Duke University School of Medicine Durham NC; ^13^ Department of Population Health Sciences Duke University School of Medicine Durham NC; ^14^ Emory Healthcare Patient and Family Advisory Council Atlanta GA; ^15^ Department of Emergency Medicine University of Michigan Medical School Ann Arbor MI; ^16^ Department of Neurology California Pacific Medical Center San Francisco CA

**Keywords:** acute myocardial infarction, clinical trial, ethics, informed consent, stroke, Ethics and Policy, Clinical Studies, Cerebrovascular Disease/Stroke, Acute Coronary Syndromes

## Abstract

**Background:**

Emergent informed consent for clinical trials in acute myocardial infarction (AMI) and stroke is challenging. The role and value of consent are controversial, and insufficient data exist regarding patients’ and surrogates’ experiences.

**Methods and Results:**

We conducted structured interviews with patients (or surrogates) enrolled in AMI or acute stroke trials at 6 sites between 2011 and 2016. Primary domains included trial recall, consent experiences, and preferences regarding involvement. Descriptive and test statistics were used to characterize responses and explore relationships between key domains and characteristics. Multivariable logistic regression was used to examine associations between key covariates and consent preferences. There were 176 (84 stroke, 92 AMI) completed interviews. Most stroke respondents (82%) were surrogates; all AMI respondents were patients. Average time from trial enrollment to interview was 1.9 years (stroke) and 2.8 years (AMI); 89% of stroke and 62% of AMI respondents remembered being in the trial, and among these respondents, 80% (stroke) and 44% (AMI) remembered reading some of the consent form. Over 90% reported not feeling pressure to enroll, being treated in a caring way, and being treated with dignity. A minority (16% stroke and 26% AMI) reported they would have preferred not to be asked for consent. Just over half (61% stroke and 53% AMI) recalled a postenrollment conversation about the study.

**Conclusions:**

Most respondents felt they were treated respectfully and were glad they had been asked for consent. Trial recall was relatively low, and many respondents recalled little postenrollment discussion. Further development of context‐sensitive approaches to consent is important.

**Clinical Perspective**

**What Is New?**

- Despite known barriers to consent for clinical trials in acute cardiovascular conditions, patients and surrogates interviewed in this study generally felt respected by consent processes and were glad they were asked for consent across a range of clinical trials for acute myocardial infarction and acute stroke.

**What Are the Clinical Implications?**

- Expectations of consent for clinical trials in acute cardiovascular conditions need to be contextualized, and there is a need for development, refinement, and further testing of approaches to consent that take the acuity of the clinical situation into account.

## Introduction

The overarching goals of informed consent for clinical research are to respect and protect participants, to inform them about key aspects of the study, and to provide them with an opportunity to participate or decline.^1^ How to advance these goals in the context of medical emergencies such as acute myocardial infarction (AMI) and stroke is unclear. In both situations, either a capacitated patient or surrogate is typically present. However, decisions about treatment and trial enrollment must be made rapidly, often in the context of acute symptoms and substantial stress.^2^

Regulations in the United States and other countries permit clinical trials to be conducted under an exception from informed consent in certain emergency settings.^3^, ^4^ Some trials in myocardial infarction (MI) and stroke have used these mechanisms. Examples include the HEAT‐PPCI (Unfractionated Heparin Versus Bivalirudin in Primary Percutaneous Coronary Intervention) trial in ST‐elevation myocardial infarction and the FAST‐MAG (Field Administration of Stroke Therapy‐Magnesium) trial.^5^, ^6^ Other studies, such as the TASTE‐MI (Thrombus Aspiration in ST‐Elevation Myocardial Infarction) trial, have utilized a brief verbal consent process.^7^ However, acute stroke and AMI trials, especially in the United States, have typically been conducted using standard informed consent processes, often including long forms containing all elements required by Food and Drug Administration and Department of Health and Human Services regulations.^8^, ^9^ These regulations require 6 basic elements and up to 9 additional elements. Determining the necessary detail is left to the discretion of institutional review boards (IRBs).

The appropriateness of standard consent in AMI and acute stroke trials is unclear. Limited available data suggest these patients and surrogates do often value being involved in trial enrollment decisions prospectively.^10^, ^11^, ^12^, ^13^, ^14^ However, they also suggest that patients and surrogates have a minimal understanding of the study at enrollment. The latter finding is not surprising given time constraints and frequent use of medications (eg, sedation for procedures) that could affect cognition or memory. Unfortunately, little is known regarding the quality of these patients’ and surrogates’ experiences with consent, particularly whether they feel respected and appropriately involved, and what information they find important at the time of the decision. Studying experiences of patients and surrogates in trials for acute stroke and AMI can promote development of approaches that simultaneously advance respect for persons, acknowledge the reality of emergency settings, and facilitate important trials. This interview study was designed to identify practical opportunities for improvement and alignment of these goals.

## Methods

The P‐CARE (Patient‐Centered Approaches to Research Enrollment Decisions in Acute Cardiovascular Disease) study was a cross‐sectional interview study of individuals who made enrollment decisions across a range of clinical trials for ST‐elevation MI and acute stroke. We recruited patients and surrogates of patients enrolled in trials between 2011 and 2016 at 6 US institutions selected by invitation with attention to geographic variation. This study was reviewed and approved by the Emory University Institutional Review Board and at participating sites via reliance agreement or the local IRB. Deidentified data supporting the findings of this study may be made available from the corresponding author on reasonable request for purposes of reproduction of results.

Included trials involved randomization and required enrollment in the acute phase (<24 hours from presentation). Study interventions could be procedural or medical. The individual recruited for this study was whoever made the initial enrollment decision. This was either the patient or a surrogate (legally authorized representative). All enrollees were eligible for this study unless they did not speak English. Eligible individuals were informed about P‐CARE by phone or in writing by the primary study site and referred to the P‐CARE team unless they did not want to be contacted. Full oral informed consent was obtained at the time of the interview, and participants were paid $20 for participation.

Telephone interviews were conducted by a contracted research firm (APCO Insight, Washington, DC) or a trained coordinator (A.M. or C.S.) between 2015 and 2017. Interviews used a structured guide including the following domains: clinical context in which the enrollment decision was made; trial recall; recall of the consent process; perceptions of the consent process; and preferences regarding involvement in the enrollment decision. Several validated scales were utilized. The Research Attitudes Questionnaire and a 1‐question screen for health literacy were used to characterize baseline attitudes toward research and level of health literacy.^15^, ^16^ The Decisional Regret Scale^17^ and low‐literacy Decisional Conflict Scale^18^ were components of the assessment of participants’ experiences with the enrollment decision. Demographic data, including age, sex, race, education, and marital status, were collected during the interview. Clinical data from the parent trial were unavailable for analysis. Questions were primarily closed‐ended, using Likert scales where appropriate. Open‐ended, structured follow‐up probes explored participants’ reasons for their answers.

The interview guide was created by the investigator team, which included professionals with expertise in clinical cardiology, neurology, and emergency medicine, trial conduct, qualitative and quantitative interview design, research ethics, and research regulations. The P‐CARE patient advisory panel and contracted survey firm (APCO Insight) also participated in the construction of the interview guide. Where possible, validated instruments were incorporated. Domains related to perceptions of respect were derived from prior qualitative work regarding patients’ perspectives of respectful treatment in the context of emergency clinical research.^19^, ^20^ The interview guide was cognitively pretested using the think‐aloud method and revised accordingly.

Interviews were audiorecorded, and real‐time data entry with computer‐assisted telephone interviewing was utilized for closed‐ended questions. Audio files were reviewed by a coordinator (A.M. or C.S.), and data entry was verified. Answers to open‐ended questions, structured probes, and “other” answers (without predefined responses) were coded by a coordinator using codes established by the research team. For each question for which open‐ended coding was necessary, the team created a set of a priori codes based on expected responses and the nature of the question. The coding scheme for each question was modified inductively during review of the data, consistent with the template analytic method.^21^ All open‐ended data were reviewed using the final codebook. All “other” answers and any answers for which the primary coder was uncertain were reviewed by 3 authors (C.S., A.M., and N.D.). Discrepancies were resolved by consensus.

### Statistical Analysis

Frequency (percentage) and mean (with standard deviation) were used to summarize categorical and continuous variables for survey items and demographic criteria. Chi‐squared, Fisher exact, and ANOVA tests were used to characterize responses and explore relationships among key domains, the condition under study, and other potential predictor variables. Multivariable logistic regression was used to examine the relationship between key covariates (condition, age, sex, race, health literacy, and decisional uncertainty) and questions related to consent preferences. Analyses were conducted using SAS 9.4 (Cary, NC). For all analyses, *P*≤0.05 was considered statistically significant.

## Results

### Study Population

Of 540 individuals referred, 197 (102 stroke and 95 AMI) provided consent to be interviewed (Figure, response rate=36%). Interviews were excluded for 21 participants because it was determined (during the interview or on review) that the respondent was not the primary decision maker (n=17) or the trial enrollment decision was not in the acute phase (n=4).

**Figure 1:**
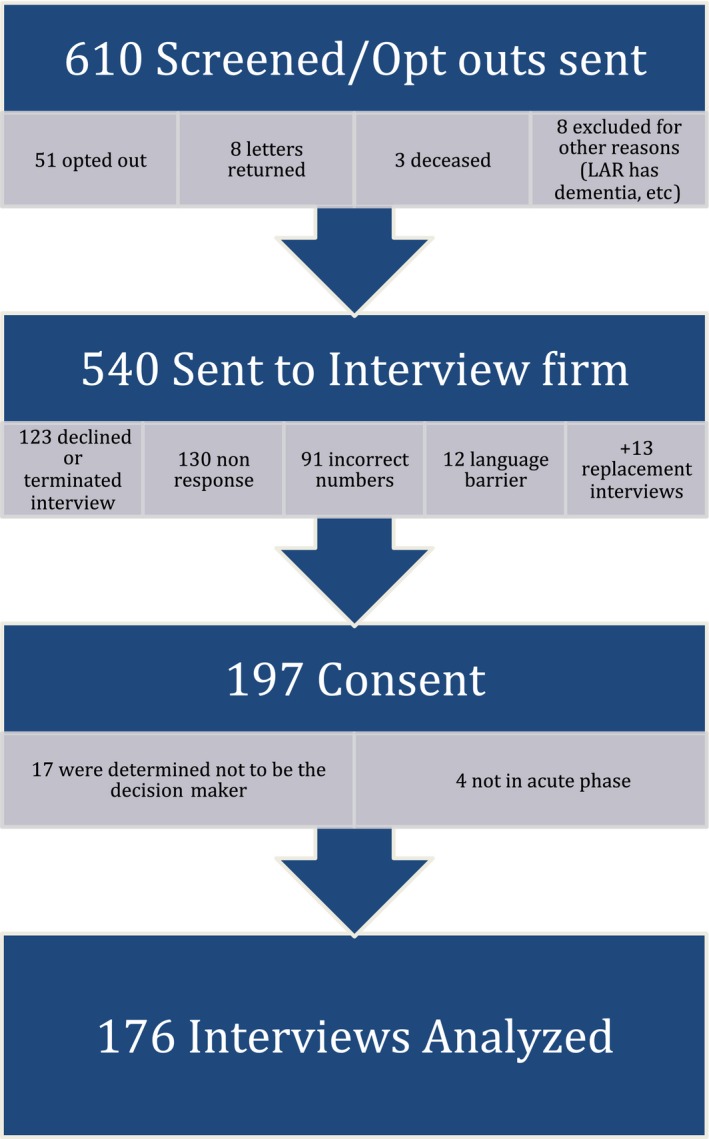
Study enrollment.

Respondents came from 6 sites. Stroke respondents were recruited from 10 trials across 3 sites. AMI respondents were recruited from 7 trials across 4 sites. One site referred both stroke and AMI respondents (Table 1). Approximately half (51%) of stroke respondents were involved in a trial testing medical management, 27% in a trial of a novel device, and 21% in a trial of a procedural intervention. Among AMI respondents, 49% were enrolled in a trial of a procedural intervention, 47% in a trial of medical management, and 4% in a trial of a novel device. The average time from enrollment to interview was 1.9 (SD 1.3) years and 2.8 (SD 1.2) years among stroke and AMI respondents, respectively.

**Table 1:** Trial Names

	Clinicaltrials.gov ID
Stroke^a^	
ATACH‐II (Antihypertensive Treatment of Acute Cerebral Hemorrhage‐II)	NCT01176565
CLEAR III (Clot Lysis: Evaluating Accelerated Resolution of Intraventricular Hemorrhage Phase III)	NCT00784134
Evaluation of Cerebral Edema in Acute Ischemic and Hemorrhagic Stroke Using Volumetric Integral Phase‐shift Spectroscopy: a Pilot Study	N/A
DAWN (Clinical Mismatch in the Triage of Wake Up and Late Presenting Strokes Undergoing Neurointervention With Trevo)	NCT02142283
MISTIE III (Minimally Invasive Surgery Plus Rt‐PA for ICH Evacuation Phase III)	NCT01827046
A Randomized, Concurrent Controlled Trial to Assess the Safety and Effectiveness of the Separator 3D as a Component of the Penumbra System in the Revascularization of Large Vessel Occlusion in Acute Ischemic Stroke	NCT01584609
RHAPSODY (Safety Evaluation of 3K3A‐APC in Ischemic Stroke)	NCT02222714
SWIFT PRIME (Solitaire With the Intention for Thrombectomy as Primary Endovascular Treatment Trial)	NCT01657461
TREVO2 (Randomized Trial Evaluating Performance of the Trevo Retriever Versus the Merci Retriever in Acute Ischemic Stroke)	NCT01270867
Ongoing Trial of Medical Management in Acute Stroke (name withheld due to ongoing enrollment)	Not provided due to ongoing enrollment
Acute MI	
APPOSITION V (Stentys Coronary Stent System Clinical Trial in Patients With Acute Myocardial Infarction)	NCT01732341
CRISP‐AMI (Counterpulsation Reduces Infarct Size Pre‐PCI for AMI)	NCT00833612
Pharmacological Effects of Crushing Prasugrel in STEMI Patients	NCT02212028
ICE T‐TIMI 49 (A Safety/Efficacy Study of Intracoronary Tenecteplase During Balloon Angioplasty to Treat Heart Attacks)	NCT00604695
The INFUSE—Anterior Myocardial Infarction (AMI) Study	NCT00976521
High Ticagrelor Loading Dose in STEMI	NCT01898442
TOTAL (A Trial of Routine Aspiration Thrombectomy With Percutaneous Coronary Intervention (PCI) Versus PCI Alone in Patients With ST‐Segment–Elevation Myocardial Infarction (STEMI) Undergoing Primary PCI)	NCT01149044

AMI indicates acute myocardial infarction; STEMI, ST‐segment–elevation myocardial infarction.

a Includes all trials closed to enrollment.

Of 176 respondents, the mean age was 58.8 (SD=11.6) years. Forty‐three percent were female, 64% were white, and 26% were black (Table 2). On the single‐question health literacy screen, about half (49%) of those enrolled felt “extremely confident” filling out medical forms by themselves; just under one third (29%) were “somewhat confident” or less.

**Table 2:** Demographics and Patient Characteristics (n=176)

	Stroke (n=84) N (%) or Mean (SD)	AMI (n=92) N (%) or Mean (SD)	Overall (n=176) N (%) or Mean (SD)
Age	57 (13.7)	59.7 (9.4)	58.8 (11.6)
Male	32 (38.1)	68 (73.9)	100 (56.8)
Race			
American Indian/Alaska Native	1 (1.2)	2 (2.2)	3 (1.7)
Asian	4 (4.8)	4 (4.4)	8 (4.6)
Black	28 (33.3)	17 (18.5)	45 (25.6)
Hawaiian/Pacific Islander	1 (1.2)	0	1 (0.6)
White	49 (58.3)	64 (69.6)	113 (64.2)
Multirace	0	3 (3.3)	3 (1.7)
Other	1 (1.2)	2 (2.2)	3 (1.7)
Hispanic Ethnicity	2 (2.4)	1 (1.1)	3 (1.7)
Education level			
High school or less	30 (35.7)	39 (42.4)	69 (39.2)
Some college	24 (28.6)	25 (27.2)	49 (27.8)
College or more	30 (35.7)	28 (30.4)	58 (33.0)
Employment status			
Full‐time	37 (44.1)	30 (32.6)	67 (38.1)
Part‐time	5 (6.0)	6 (6.5)	11 (6.3)
Unemployed	7 (8.3)	4 (4.4)	11 (6.3)
Retired	22 (26.2)	32 (34.8)	54 (30.7)
Disabled	9 (10.7)	16 (17.4)	25 (14.2)
Other	3 (3.6)	3 (3.3)	6 (3.4)
Missing	1 (1.2)	1 (1.1)	2 (1.1)
Marital status			
Married	53 (63.1)	53 (57.6)	106 (60.2)
Single	10 (11.9)	13 (14.1)	23 (13.1)
Divorced or separated	11 (13.1)	16 (17.4)	27 (15.3)
Unmarried living with partner	0	4 (4.4)	4 (2.3)
Widow or widower	9 (10.7)	5 (5.4)	14 (8.0)
Missing	1 (1.2)	1 (1.1)	2 (1.1)
Interviewees			
Patient	15 (17.9)	92 (100)	107 (60.8)
Surrogate	69 (82.1)	0	69 (39.2)
Health literacy: how confident are you filling out medical forms by yourself?			
Extremely	38 (50.7)	26 (45.6)	64 (48.5)
Quite a bit	15 (20.0)	15 (26.3)	30 (22.7)
Somewhat	13 (17.3)	8 (14.0)	21 (15.9)
A little bit	8 (10.7)	5 (8.8)	13 (9.9)
Not at all	1 (1.3)	3 (5.3)	4 (3.0)
Trial type			
Medical management	43 (51.2)	43 (46.7)	86 (48.9)
Novel device	23 (27.4)	4 (4.3)	27 (15.3)
Procedural intervention	18 (21.4)	45 (48.9)	63 (35.8)
Time from trial enrollment to interview, y	1.9 (1.3)	2.8 (1.2)	2.4 (1.3)

AMI indicates acute myocardial infarction.

There were differences between stroke and AMI trial respondents. Most notably, the majority of stroke respondents (82%) were surrogate decision makers; all AMI respondents were patients. A larger number of stroke respondents were black (33% versus 19%) and female (62% versus 26%).

### Study Recall

Interviews began with an assessment of whether the respondent remembered being a part of the study in which he or she was enrolled (Table 3). Sixteen stroke respondents (19%) and 40 AMI respondents (44%) did not initially recall being a part of the study. Of the remaining participants, only 18% (stroke) and 6% (AMI) spontaneously recalled that the study involved a comparison between different treatments; 34% (stroke) and 27% (AMI) spontaneously recalled that the study was investigating a treatment. Most of the others in both groups either thought the study involved data collection only or were uncertain what it involved. There was no significant association between time from enrollment to interview and the respondents’ level of recall of the study (stroke *P*=0.338; MI *P*=0.262). A total of 75 stroke respondents and 57 AMI respondents had sufficient recall of participation (n=132) to be asked subsequent questions regarding their experiences related to consent and participation.

**Table 3:** Patients’ and Surrogates’ Recall of Stroke and AMI Trials

Recall of Being in a Study	Stroke, N=84 n (%)	AMI, N=92 n (%)	*P* Value
Do you remember the patient/you being a part of any research studies when you/he/she were treated at [SITE] in [YEAR]?			
Yes	67 (79.8)	44 (47.8)	<0.001 a
No	16 (19.1)	40 (43.5)	
Don't know	1 (1.2)	8 (8.7)	
Can you explain to me what that research study was about (Asked if “Yes” or “Don't know” above)?^b^			
Comparison of different treatments	12 (17.7)	3 (5.8)	0.01 a
Studying a treatment	23 (33.8)	14 (26.9)	
Data collection only	17 (25.0)	7 (13.5)	
Incorrect study recall	1 (1.5)	4 (7.7)	
Don't know	13 (19.1)	20 (38.5)	
No answer^c^	2 (2.9)	4 (7.7)	
Do you remember [PATIENT]/you being a part of that study or hearing about a study like that at any point (all respondents)?			
Yes	75 (89.3)	57 (62.0)	<0.001
No	5 (6.0)	29 (31.5)	
Don't know	4 (4.8)	6 (6.5)	

	Stroke, N=75 n (%)	AMI, N=57 n (%)	*P* Value
Recollections of study details			
What did you think were the potential benefits to the patient/for you being a part of this study (multiple answers allowed)?			
Benefit to self/patient	40 (51.3)	14 (23.0)	0.007^a^
Help others/contribute to research	14 (18.0)	22 (36.1)	
More education on the condition	7 (9.0)	3 (4.9)	
Improved care/monitoring	4 (5.1)	3 (4.9)	
No benefit	2 (2.6)	3 (4.9)	
Don't know	8 (10.3)	8 (13.1)	
No answer^c^	3 (3.9)	8 (13.1)	
Do you remember being told about any risks associated with being included in this study?			
Yes	29 (38.7)	19 (33.3)	0.43
No	37 (49.3)	34 (59.7)	
Don't know	9 (12.0)	4 (7.0)	
Reasons for joining the study			
What was the main reason why you decided to include the patient/join the study?			
Benefit to self/patient	38 (50.7)	9 (15.8)	<0.001 a
Trust in doctor	2 (2.7)	1 (1.8)	
Absence of risk	6 (8.0)	2 (3.5)	
Help others/contribute to research	19 (25.3)	29 (50.9)	
More education on the condition	3 (4.0)	2 (3.5)	
Improved care/monitoring	0 (0)	2 (3.5)	
Other	4 (5.3)	4 (7.0)	
Don't know	2 (2.7)	4 (7.0)	
No answer^c^	1 (1.3)	4 (7.0)	

AMI indicates acute myocardial infarction.

a Fisher exact test, all other tests are chi‐squared.

b Responses are missing because not all participants were asked this question due to a skip pattern.

c Responses are missing due to participants not providing an answer.

Approximately one third of patients in each group remembered being told about risks associated with the trial in which they were included. The groups differed significantly in what they perceived to be the potential benefits of participation: 51% of stroke respondents versus 23% of AMI respondents reported a potential for direct benefit. In contrast, 36% of AMI respondents versus 18% of stroke respondents thought the potential benefit was helping others or contributing to research. This is consistent with the fact that 51% of stroke respondents (versus 16% of AMI respondents) stated that the main reason for joining the study was a chance for benefit. In contrast, 51% of AMI respondents (versus 25% of stroke respondents) stated that the main reason for joining the study was a desire to contribute to research.

### Clinical Context

Significant differences were observed in reported level of emotional stress at the time of the enrollment decision: 39 (42%) AMI respondents versus 12 (14%) stroke respondents rated their level of stress as a 5 or less on a 10‐point scale, with 10 representing “extremely stressed.” Similarly, when asked to rate their level of discomfort or severity of symptoms (of the patient for stroke respondents), 42 (46%) AMI respondents compared with 6 (7%) stroke respondents rated discomfort/severity as 5 or less.

### Experience of Consent

AMI respondents reported significantly shorter times for the consent conversation (time it took to describe the study) and a trend toward shorter time to make the enrollment decision (Table 4). AMI respondents were also less likely to report asking questions at the time of enrollment (30% versus 60%). These differences are consistent with the fact that ST‐segment–elevation MI guidelines dictate strict timeframes, whereas stroke interventions tested were more heterogeneous, and the decision maker was almost always a surrogate.

**Table 4:** Experiences With Consent

Recollection of Consent Process	Stroke N=75 n (%)	AMI N=57 n (%)	*P* Value
How long do you remember [the person who described the study] taking to describe the study to you (min)? (Stroke, n=67; MI, n=47)^a^			
<5 min	12 (17.9)	18 (38.3)	0.002^b^
>5 and <10 min	12 (17.9)	5 (10.6)	
>10 and <15 min	15 (22.4)	9 (19.2)	
>15 min	25 (37.3)	6 (12.8)	
Do not know	2 (3.0)	7 (14.9)	
No answer^c^	1 (1.5)	2 (4.3)	
How long did it take for you to make a decision about whether to join the study?			
<5 min	47 (62.7)	46 (80.7)	0.06^b^
>5 and <15 min	18 (24.0)	8 (14.0)	
>15 and <30 min	4 (5.3)	0	
>30 min	6 (8.0)	2 (3.5)	
Don't know	0	1 (1.8)	
Do you remember asking any questions about the [INSERT STUDY NAME] study?			
Yes	45 (60.0)	17 (29.8)	<0.001^b^
No	25 (33.3)	38 (66.7)	
Don't know	5 (6.7)	1 (1.8)	
No answer^c^	0	1 (1.8)	

Recollection of Consent Form	Stroke N=65 n (%)	MI N=43 n (%)	*P* Value
Did you read the consent form right then, before you signed it? a			
Yes	52 (80.0)	19 (44.2)	<0.001^b^
No	9 (13.9)	22 (51.2)	
Don't know	4 (6.2)	2 (4.7)	
Which statement best describes how much of the consent form you read prior to signing it?^a^ (Stroke n=52; MI n=19)^a^			
I read or skimmed a little bit of it	8 (15.4)	2 (10.5)	0.77^b^
I read or skimmed through most key parts	22 (42.3)	11 (57.9)	
I read the full document	20 (38.5)	6 (31.6)	
Don't know	2 (3.9)	0	

Interaction With Study Staff	Stroke N=75 n (%)	AMI N=57 n (%)	*P* Value
After the patient/you were enrolled in the study, did anyone talk with you about what the study involved?			
Yes	46 (61.3)	30 (52.6)	0.36^b^
No	19 (25.3)	15 (26.3)	
Don't know	10 (13.3)	10 (17.5)	
No answer^c^	0	2 (3.5)	
When talking to me about the study, the person who asked me to sign the consent form seemed to understand how I was feeling at the time			
Agree	65 (86.7)	49 (86.0)	0.91^b^
Not agree^d^	8 (10.7)	7 (12.3)	
No answer^c^	2 (2.7)	1 (1.8)	
The person who talked to me about the study acted in a caring way toward me			
Agree	72 (96.0)	51 (89.5)	0.27^b^
Not agree^d^	2 (2.7)	5 (8.8)	
No answer^c^	1 (1.3)	1 (1.8)	
The person who talked to me about the study explained how the study related to the patient/my medical situation			
Agree	67 (89.3)	41 (71.9)	0.02^b^
Not agree^d^	7 (9.3)	15 (26.3)	
No answer^c^	1 (1.3)	1 (1.8)	
The person who talked to me about the study treated me with dignity			
Agree	73 (97.3)	53 (93.0)	0.02^b^
Not agree^d^	0	4 (7.0)	
No answer^c^	2 (2.7)	0	
I felt pressure from doctors or other people to include the patient/be included in the study			
Agree	3 (4.0)	3 (5.3)	1.00^b^
Not agree^d^	72 (96.0)	54 (94.7)	
[The person who described the study] told me everything I needed to know about the [INSERT STUDY NAME] study			
Agree	67 (89.3)	41 (73.2)	0.02^b^
Not agree^d^	7 (9.3)	15 (26.8)	
No answer^c^	1 (1.3)	1 (1.8)	

AMI indicates acute myocardial infarction; MI, myocardial infarction.

a Responses are missing because not all participants were asked this question due to a skip pattern.

b Fisher exact test, all else is chi‐squared.

c No answer implied participants either did not provide an answer or were not asked the question.

d Includes those who responded “Don't know.”

Most respondents remembered signing a consent form (87% stroke and 75% AMI). Stroke respondents, however, were significantly more likely to report having read the form before signing it (80% versus 44%), with the most common answer in both groups being to “read or skim through most key parts.” Just over half the respondents in both groups (61% for stroke and 53% for AMI) reported remembering anyone talking to them later about the study.

Reported decisional uncertainty was low in both groups (stroke 8.3 [0‐25]; AMI 0 [0‐33.33]; median summed score for each), and decisional regret was low (stroke: 0 [0‐0]; AMI 0 [0‐10]; median summed score for each). The large majority in both groups felt they were given the right amount of information before deciding whether to join the study (80% stroke and 72% AMI), although the most common answers to what information was most valuable to them were different. Stroke participants most often stated that benefit to the patient was most important; AMI participants most often stated that the opportunity to help others was most important.

The large majority in both groups had a favorable view of the person who had asked them for consent (Table 4) and reported being treated with empathy and dignity and that the person asking for consent acted in a caring way. AMI respondents were less likely to report that that person told them everything they needed to know about the study (73% versus 89%) and that the person asking for consent “explained how the study related to the patient/my own medical situation (72% versus 89%).”

### Views and Preferences of the Process

Almost all (100% stroke and 91% AMI) respondents felt that they were able to make the decision about whether to be a part of the study, and over 90% in both groups felt that they would have been free to decline participation if they had wanted to do so. Fewer than 10% in both groups reported having felt pressured to be in the study (Table 4).

Respondents were asked their thoughts on the most appropriate approaches to consent (Table 5), and 92% of stroke and 83% of AMI respondents were glad that they had been asked for permission before being included in the study. However, 16% of stroke and 26% of AMI respondents did state that they would have preferred that the doctor treating them had made the decision about enrollment and asked them later for consent to stay in the study. In multivariable analysis only lower decisional certainty (odds ratio 0.18 [0.07‐0.43], *P*<0.001) was significantly associated with a lower preference for being asked for consent. Regarding the consent form itself, 17% of stroke and 35% of AMI respondents wished that they had not had to sign a consent form at the time. In multivariable analysis nonwhite race (odds ratio 6.92 [1.64‐29.15], *P*=0.008) and greater decisional uncertainty (odds ratio 2.80 [1.60‐4.90], *P*<0.001) were associated with a preference for not having to sign a consent form. Greater health literacy was associated with a lower preference (odds ratio 0.32 [0.11‐0.93], *P*=0.036) for not having to sign a consent form. Fifty‐two perecnt of stroke respondents and 33% of AMI respondents, respectively, thought it was important to read written information at the time; there were no significant predictors of response to this question.

**Table 5:** Views and Preferences Regarding the Consent Process

Views and Preferences of the Process	Stroke, N=75 n (%)	AMI, N=57 n (%)	*P* Value
I am glad that I was asked before the patient was included/being included in the study at the time the patient/I was being treated for his/her/my heart attack/stroke			
Agree	69 (92.0)	47 (82.5)	0.04^b^
Not agree a	3 (4.0)	9 (15.8)	
No answer^c^	3 (4.0)	1 (1.8)	
I wish that I had not had to sign a consent form when I was asked to include the patient/be a part of the study while I/the patient was being treated for my heart attack/stroke			
Agree	13 (17.3)	20 (35.1)	0.06^b^
Not agree a	58 (77.3)	35 (61.4)	
No answer^c^	4 (5.3)	2 (3.5)	
Instead of asking me before including me/the patient in the study, I would have preferred it if the doctor treating me/the patient had made the decision for me about including me/the patient in the study and asked me later whether I/the patient wanted to stay in the study			
Agree	12 (16.0)	15 (26.3)	0.22^b^
Not agree^a^	62 (82.7)	40 (70.2)	
No answer^c^	1 (1.3)	2 (3.5)	
It was important for me to read written information about the study at the time I/the patient was having a heart attack/stroke and was deciding to be a part of the study			
Agree	39 (52.0)	19 (33.3)	0.07^b^
Not agree a	35 (46.7)	36 (63.2)	
No answer^c^	1 (1.3)	2 (3.5)	

AMI indicates acute myocardial infarction.

a Includes those who responded “don't know.”

b Fisher exact test, all else is chi‐squared.

c No answer implied participants either did not provide an answer or when not asked the question.

## Discussion

This study is the most comprehensive attempt to date to capture experiences of patients and decision makers for patients enrolled in acute stroke and AMI trials where clinical treatment and enrollment decisions must happen very quickly. Debates over the proper approach to consent have simmered for years, but there have been few attempts to study these individuals’ experiences or to design consent processes to match the contexts in which these trials take place. The findings from this study demonstrate the value of involving patients and surrogates prospectively in enrollment decisions in acute settings, as well as the complexity and limitations intrinsic to these clinical contexts. These findings have important practical implications.

First, it is encouraging that most participants’ consent experiences were positive. Some have argued that involving patients and surrogates in trial enrollment decisions in acute settings is inappropriate and insensitive to the stress and fear intrinsic to these situations.^22^ However, this study reinforces prior findings that suggest individuals generally prefer prospective involvement as opposed to enrollment under an exception from informed consent or deferred consent.^10^ It is particularly important that respondents reported high rates of being treated with dignity and being treated in a caring and empathic manner as well as very low decisional regret.

These findings, also help to illustrate that consent processes play multiple roles. Although the focus of informed consent is generally on facilitating an informed, voluntary decision, consent is rooted in the principle of respect for persons, which extends beyond respect for autonomy and incorporates treating individuals with dignity and empathy.^2^, ^23^ Consent processes can also help to advance transparency and allow people to exert control over enrollment, even in the absence of substantial understanding of a trial.^23^, ^24^ More pragmatically, positive experiences with consent in stressful, emergent situations may help to sustain (and not undermine) trust in medical research. For all of these reasons, we believe this study suggests that there is value for participants in being involved in some form of consent, even when understanding is poor, as in many AMI trials. Abandoning prospective involvement altogether seems potentially counterproductive and unnecessary.

Second, the data confirm what most acute care researchers already know—that decisions are made quickly and that understanding of trials in acute contexts is likely minimal. Although the time between trial enrollment and the interview was lengthy in many cases, this length of time was not significantly related to recall accuracy. This is consistent with other studies’ findings that low understanding and recall are common, even when assessed shortly after consent.^25^, ^26^ Many individuals were also not aware that the study in which they were enrolled (or in which they had enrolled the patient) was studying an intervention. In all these ways, our findings illustrate that expectations of fully informed decisions in stroke trials, and especially in AMI trials, are unrealistic. Similarly, long, detailed consent forms that are the norm seem inappropriate and unhelpful. Relatively low numbers of participants reported reading the entire form before enrollment, and among our respondents, blacks and individuals with lower health literacy had particularly unfavorable views of consent forms.

Third, very important differences were observed between the 2 populations of respondents within this study. Specifically, decision timeframes were longer, surrogates were more likely to be the decision maker, recall of the study was greater, and reports of reading at least some portion of consent forms were greater among stroke respondents. These differences are likely driven by the fact that decision makers were surrogates and that some stroke trials had less pronounced time constraints on enrollment decisions than others. These findings suggest that there may be more opportunity for engagement in stroke trials. Written materials especially may have more potential to help inform decision makers for stroke trials than AMI trials. The data suggest, however, that consent processes (and forms) should be short, clear, and focused on what matters most to decision makers in both groups.

In light of these findings this study offers several practical avenues for improvement. Most concretely, it suggests a need for simple, short, and clear consent processes. P‐CARE investigators and patient advisors have developed model forms (http://www.eccri.emory.edu/research/ethics/index.html), but it will require testing within actual trials to determine how much consent forms or other media can improve communication in this setting and what information individuals most want to know in emergent conditions. At a minimum, these data provide a strong case for using materials that mirror actual conversations, avoid information overload, and are of a length and complexity that fit the trial context. It is possible that the newly revised Common Rule requirement for “concise and focused presentation of key information” at the beginning of consent forms may be helpful in this effort, although it is not clear that the revised Common Rule, when implemented, will result in shorter or simpler consent forms in general.^27^

A fourth practical implication concerns the need for communication with patients and surrogates after initial enrollment. In addition to recalling relatively few details about the trial, a substantial number of respondents did not recall anyone talking with them about the study after enrollment had taken place. It may be that these discussions took place but that their recollection of postenrollment conversations was poor. However, attention to postenrollment communication could be valuable. It could facilitate decisions about subsequent components of the study. Potentially more importantly, it could help individuals to understand the activity in which they are involved, to feel respected, and to appreciate the important contribution they are making as research participants. This may help to increase awareness and familiarity with research generally.

A final implication of this work is regulatory in nature. It remains to be seen what strategies most appropriately and effectively involve participants and surrogates in these decisions. It also remains to be seen whether highly abbreviated consent processes will be considered by IRBs to meet regulatory standards. Food and Drug Administration and Department of Health and Human Services regulations provide little specificity regarding how detailed consent processes need to be regarding the required elements. IRBs must thus determine whether informed consent documents and processes are appropriate for the context of the trial, the elements of the trial, and the study population. Brief, targeted consent processes and forms tailored to the acute context with sufficient detail to meet regulatory requirements are important to consider and appear to have some public support.^28^ We believe this approach is consistent with the “reasonable person standard” invoked by the new revised Common Rule and may be advanced by the requirement to present concise and focused key information upfront.^27^ However, whether IRBs will approve more targeted consent forms is uncertain. If context‐appropriate solutions cannot be developed and approved within standard regulations, the exception from informed consent regulations may offer an alternative path forward. Studies such as the IMMEDIATE (Immediate Myocardial Metabolic Enhancement During Initial Assessment and Treatment in Emergency Care) trial in the prehospital setting, for example, have been conducted under the exception from informed consent while they still utilize an assent that retains an element of participant involvement.^29^

### Limitations

Although it is larger and more comprehensive than most prior studies of consent in AMI and stroke, this study contains important limitations. First, the length of time between enrollment and interview was substantial. We suspect that this limits the recall of trial details more than recall of the consent experience, but it may limit both and obscure distinctions between consent for clinical care and research. Embedding prospective, contemporaneous assessment into future clinical trials in AMI and acute stroke is important. Embedded empirical studies of consent also offer exciting opportunities to assess the impact of innovative approaches. Second, and closely related, we did not have any means of assessing what actually took place beyond respondents’ recall. In addition, we did not ask individuals who did not recall participation any further questions regarding consent preferences. It is possible that these individuals hold different views about consent. Third, our study was limited to individuals willing to be interviewed about their experiences; we do not have data regarding how they differed from those who did not respond. Fourth, we did not have access to patients’ clinical outcomes and cannot assess the impact of trial‐related outcomes on views of consent. Prior work has demonstrated some associations between better outcomes and more favorable views of the exception from informed consent in emergency research^30^; further investigation of these associations in stroke and MI research may help to identify post‐enrollment communication strategies. Finally, this structured interview guide facilitated only limited probing of positive and negative experiences. A companion qualitative key informant interview study was conducted with a subset of participants in order to provide greater insight into specific drivers of positive and negative experiences and to explore alternative approaches. These data will be analyzed separately.

## Conclusions

In this interview study with patients and surrogates of patients enrolled in a range of acute stroke and acute MI trials, the large majority of respondents felt that they had been treated respectfully during the enrollment decision and were glad that they had been asked for consent prospectively. However, understanding of the trial was relatively poor, many respondents did not recall substantial contact after initial enrollment occurred, and the views of consent forms were mixed. Exploration of context‐sensitive approaches to consent that incorporate highly simplified consent language, targeting of the most essential elements of informed consent, explicit attention to postenrollment contact, and recognition of the nature of decision making on the part of investigators and IRBs may help to maximize respect for participants while facilitating important studies.

## Sources of Funding

Research reported in this article was funded through a PCORI (Patient‐Centered Outcomes Research Institute) Award (ME‐1402‐10638). The views presented are solely the responsibility of the authors and do not necessarily represent the views of PCORI, its Board of Governors, or its Methodology Committee.

## Disclosures

Dr Dickert reports receiving research support from PCORI and the National Institutes of Health (NIH). Dr Adeoye reports receiving research support from NIH and the National Institute of Neurological Disease and Stroke. Dr Angiolillo reports receiving payments as an individual for: a) Consulting fee or honorarium from Amgen, Aralez, AstraZeneca, Bayer, Biosensors, Boehringer Ingelheim, Bristol‐Myers Squibb, Chiesi, Daiichi‐Sankyo, Eli Lilly, Haemonetics, Janssen, Merck, PLx Pharma, Pfizer, Sanofi, and The Medicines Company; b) Participation in review activities from CeloNova and St. Jude Medical. Institutional payments for grants from Amgen, AstraZeneca, Bayer, Biosensors, CeloNova, CSL Behring, Daiichi‐Sankyo, Eisai, Eli‐Lilly, Gilead, Janssen, Matsutani Chemical Industry Co., Merck, Novartis, Osprey Medical, and Renal Guard Solutions. Dr Angiolillo reports receiving research support from the Scott R. MacKenzie Foundation and the NIH/NCATS Clinical and Translational Science Award to the University of Florida UL1 TR000064 and NIH/NHGRI U01 HG007269. Dr Devireddy reports serving on scientific advisory boards for Medtronic, ReCor Medical, and Vascular Dynamics. Dr Nogueira reports research support from Stryker Neurovascular, Medtronic, Penumbra, and Neuravi, and he reports serving on advisory boards for Genentech, Allm Inc, and Cerenovus. Dr Parker reports receiving research support from the NIH, the FDA, and Merck. Dr Patel reports research support from Maquet, Bayer, AstraZeneca, Janssen, and Proctor and Gamble. Dr. Patel reports serving on advisory boards for Bayer and Janssen. Dr Pentz reports receiving research support from NIH. Dr Silbergleit reports research support from NIH. Dr Weinfurt reports receiving consulting fees from Regeneron.

## References

[1] National Commission for the Protection of Research Risks . The Belmont Report: Ethical Principles and Guidelines for the Protection of Human Subjects of Research. Washington, DC: Government Printing Office; 1979.

[2] Dickert NW , Brown J , Cairns CB , Eaves‐Leanos A , Goldkind SF , Kim SY , Nichol G , O'Conor KJ , Scott JD , Sinert R , Wendler D , Wright DW , Silbergleit R . Confronting ethical and regulatory challenges of emergency care research with conscious patients. Ann Emerg Med. 2016;67:538–545. 26707358 10.1016/j.annemergmed.2015.10.026 PMC7749649

[3] US Food and Drug Administration . Title 21 (Code of Federal Regulations), Part 50.24 Protection of human subjects. 2004.

[4] van Belle G , Mentzelopoulos SD , Aufderheide T , May S , Nichol G . International variation in policies and practices related to informed consent in acute cardiovascular research: results from a 44 country survey. Resuscitation. 2015;91:76–83. 25524361 10.1016/j.resuscitation.2014.11.029

[5] Saver JL , Starkman S , Eckstein M , Stratton SJ , Pratt FD , Hamilton S , Conwit R , Liebeskind DS , Sung G , Kramer I , Moreau G , Goldweber R , Sanossian N ; FAST‐MAG Investigators and Coordinators . Prehospital use of magnesium sulfate as neuroprotection in acute stroke. N Engl J Med. 2015;372:528–536. 25651247 10.1056/NEJMoa1408827 PMC4920545

[6] Shahzad A , Kemp I , Mars C , Wilson K , Roome C , Cooper R , Andron M , Appleby C , Fisher M , Khand A , Kunadian B , Mills JD , Morris JL , Morrison WL , Munir S , Palmer ND , Perry RA , Ramsdale DR , Velavan P , Stables RH ; for the HEAT‐PPCI Trial Investigators . Unfractionated heparin versus bivalirudin in primary percutaneous coronary intervention (HEAT‐PPCI): an open‐label, single centre, randomised controlled trial. Lancet. 2014;384:1849–1858. 25002178 10.1016/S0140-6736(14)60924-7

[7] Frobert O , Lagerqvist B , Olivecrona GK , Omerovic E , Gudnason T , Maeng M , Aasa M , Angeras O , Calais F , Danielewicz M , Erlinge D , Hellsten L , Jensen U , Johansson AC , Karegren A , Nilsson J , Robertson L , Sandhall L , Sjogren I , Ostlund O , Harnek J , James SK . Thrombus aspiration during ST‐segment elevation myocardial infarction. N Engl J Med. 2013;369:1587–1597. 23991656 10.1056/NEJMoa1308789

[8] Title 45, CFR (Code of Federal Regulations), Part 46. 2009. 11686173

[9] U.S. Food and Drug Administration . Title 21 (Code of Federal Regulations), Part 50. Protection of human subjects. 2012.

[10] Dickert NW , Hendershot KA , Speight CD , Fehr AE . Patients’ views of consent in clinical trials for acute myocardial infarction: impact of trial design. J Med Ethics. 2017;43:524–529. 28039285 10.1136/medethics-2016-103866

[11] Dickert NW , Fehr AE , Llanos A , Scicluna VM , Samady H . Patients’ views of consent for research enrollment during acute myocardial infarction. Acute Card Care. 2015;17:1–4. 25555022 10.3109/17482941.2014.994642

[12] Gammelgaard A , Rossel P , Mortensen O ; in collaboration with the DANAMI‐2 Investigators . Patients’ perceptions of informed consent in acute myocardial infarction research: a Danish study. Soc Sci Med. 2004;58:2313–2324. 15047087 10.1016/j.socscimed.2003.08.023

[13] Gammelgaard A , Mortensen O , Rossel P ; in collaboration with the DANAMI‐2 Investigators . Patients’ perceptions of informed consent in acute myocardial infarction research: a questionnaire based survey of the consent process in the DANAMI‐2 trial. Heart. 2004;90:1124–1128. 15367504 10.1136/hrt.2003.021931 PMC1768493

[14] Kleindorfer D , Lindsell CJ , Alwell K , Woo D , Flaherty ML , Eilerman J , Khatri P , Adeoye O , Ferioli S , Kissela BM . Ischemic stroke survivors’ opinion regarding research utilizing exception from informed consent. Cerebrovasc Dis. 2011;32:321–326. 21921594 10.1159/000328815 PMC3712812

[15] Chew LD , Griffin JM , Partin MR , Noorbaloochi S , Grill JP , Snyder A , Bradley KA , Nugent SM , Baines AD , Vanryn M . Validation of screening questions for limited health literacy in a large VA outpatient population. J Gen Intern Med. 2008;23:561–566. 18335281 10.1007/s11606-008-0520-5 PMC2324160

[16] Rubright JD , Cary MS , Karlawish JH , Kim SY . Measuring how people view biomedical research: reliability and validity analysis of the Research Attitudes Questionnaire. J Empir Res Hum Res Ethics. 2011;6:63–68. 21460589 10.1525/jer.2011.6.1.63 PMC3253733

[17] Brehaut JC , O'Connor AM , Wood TJ , Hack TF , Siminoff L , Gordon E , Feldman‐Stewart D . Validation of a decision regret scale. Med Decis Making. 2003;23:281–292. 12926578 10.1177/0272989X03256005

[18] Linder SK , Swank PR , Vernon SW , Mullen PD , Morgan RO , Volk RJ . Validity of a low literacy version of the Decisional Conflict Scale. Patient Educ Couns. 2011;85:521–524. 21300518 10.1016/j.pec.2010.12.012 PMC3121898

[19] Dickert N , Kass NE . Patients’ perceptions of research in emergency settings: a study of survivors of sudden cardiac death. Soc Sci Med. 2009;68:183–191. 19004536 10.1016/j.socscimed.2008.10.001 PMC2660168

[20] Dickert NW , Kass NE . Understanding respect: learning from patients. J Med Ethics. 2009;35:419–423. 19567690 10.1136/jme.2008.027235 PMC3110664

[21] Cassell C . Template Analysis. The SAGE Dictionary of Qualitative Management Research. London, UK: SAGE Publications Ltd; 2008.

[22] Shaw D . HEAT‐PPCI sheds light on consent in pragmatic trials. Lancet. 2014;384:1826–1827. 25002175 10.1016/S0140-6736(14)61040-0

[23] Dickert NW , Miller FG . Involving patients in enrolment decisions for acute myocardial infarction trials. BMJ. 2015;351:h3791. 26223782 10.1136/bmj.h3791 PMC4707514

[24] Dickert NW , Eyal N , Goldkind SF , Grady C , Joffe S , Lo B , Miller FG , Pentz RD , Silbergleit R , Weinfurt KP , Wendler D , Kim SYH . Reframing consent for clinical research: a function‐based approach. Am J Bioeth. 2017;17:3–11. 10.1080/15265161.2017.1388448 29148951

[25] Mandava A , Pace C , Campbell B , Emanuel E , Grady C . The quality of informed consent: mapping the landscape. A review of empirical data from developing and developed countries. J Med Ethics. 2012;38:356–365. 22313664 10.1136/medethics-2011-100178 PMC4825806

[26] Nishimura A , Carey J , Erwin PJ , Tilburt JC , Murad MH , McCormick JB . Improving understanding in the research informed consent process: a systematic review of 54 interventions tested in randomized control trials. BMC Med Ethics. 2013;14:28. 23879694 10.1186/1472-6939-14-28 PMC3733934

[27] Federal policy for the protection of human subjects: final rule. Federal Register. 2017;82:7149. 28106360

[28] Dickert NW , Wendler D , Devireddy CM , Goldkind SF , Ko YA , Speight CD , Kim SYH . Consent for pragmatic trials in acute myocardial infarction. J Am Coll Cardiol. 2018;71:1051–1053. 29495987 10.1016/j.jacc.2017.12.043 PMC6151267

[29] Selker HP , Beshansky JR , Sheehan PR , Massaro JM , Griffith JL , D'Agostino RB , Ruthazer R , Atkins JM , Sayah AJ , Levy MK , Richards ME , Aufderheide TP , Braude DA , Pirrallo RG , Doyle DD , Frascone RJ , Kosiak DJ , Leaming JM , Van Gelder CM , Walter GP , Wayne MA , Woolard RH , Opie LH , Rackley CE , Apstein CS , Udelson JE . Out‐of‐hospital administration of intravenous glucose‐insulin‐potassium in patients with suspected acute coronary syndromes: the IMMEDIATE randomized controlled trial. JAMA. 2012;307:1925–1933. 22452807 10.1001/jama.2012.426 PMC4167391

[30] Whitesides LW , Baren JM , Biros MH , Fleischman RJ , Govindarajan PR , Jones EB , Pancioli AM , Pentz RD , Scicluna VM , Wright DW , Dickert NW . Impact of individual clinical outcomes on trial participants’ perspectives on enrollment in emergency research without consent. Clin Trials. 2017;14:180–186. 28359192 10.1177/1740774516677276 PMC5380144

